# Microstructural mapping of time-dependent diffusion MRI for predicting lymphovascular invasion in rectal cancer: a proof-of-concept investigation

**DOI:** 10.1186/s13244-026-02299-0

**Published:** 2026-05-14

**Authors:** Fulin Lu, Yanwan Li, Ran Wu, Kuide Li, Xiaoli Chen, Yisha Liu, Bin Luo, Meining Chen, Hang Li

**Affiliations:** 1 Department of Radiology, Sichuan Provincial People’s Hospital, School of Medicine, University of Electronic Science and Technology of China, Chengdu, China; 2 Department of Radiology, Sichuan Clinical Research Center for Cancer, Sichuan Cancer Hospital & Institute, Sichuan Cancer Center, Sichuan Cancer Hospital, University of Electronic Science and Technology of China, Chengdu, China; 3 Department of Pathology, Sichuan Provincial People’s Hospital, School of Medicine, University of Electronic Science and Technology of China, Chengdu, China; 4 Department of Gastrointestinal Surgery, Sichuan Provincial People’s Hospital, School of Medicine, University of Electronic Science and Technology of China, Chengdu, China; 5 Department of MR Scientific Marketing, Siemens Healthineers, Shanghai, China

**Keywords:** Microstructural, Diffusion MRI, Rectal cancer, Lymphovascular invasion, Predictive model

## Abstract

**Objective:**

To evaluate the value of time-dependent diffusion MRI (td-dMRI) derived microstructural parameters for predicting lymphovascular invasion (LVI) in rectal cancer.

**Materials and methods:**

Eighty-four resectable rectal cancer patients (stage T1, T2, T3a, T3b, and T4a) who underwent preoperative td-dMRI between March 2023 and June 2025 without neoadjuvant therapy were enrolled. Manual segmentation of tumors was performed by an experienced radiologist on each tumor’s largest cross-sectional area. Microstructural parameters (intracellular volume fraction (ICVF), cell diameter, extracellular diffusivity and cellularity) were fitted using the limited spectrally edited diffusion model implemented in MATLAB (MathWorks, Inc.). Apparent diffusion coefficient (ADC) values at different diffusion times, relative ADC, ADC ratio, and MRI-reported extramural vascular invasion (EMVI) were also evaluated. Mann–Whitney U test was used to evaluate parameter differences between LVI-positive and LVI-negative. Logistic regression and receiver operating characteristic (ROC) curves (with DeLong test) were used to identify predictors of LVI and diagnostic performance.

**Results:**

Of 84 participants (median age, 66 years; IQR, 60–70 years; 50 male), 30 were LVI-positive and 54 LVI-negative. ICVF, cell diameter, and cellularity were significantly higher in LVI-positive cases (all *p* < 0.05). MRI-EMVI (OR = 3.251), ICVF (OR = 8.137), and cellularity (OR = 1.159) were independent risk factors of LVI. The combined model integrating MRI-reported EMVI, cellularity, and ICVF achieved an area under the ROC curve (AUC) of 0.860, outperforming individual parameters including MRI-reported EMVI (AUC = 0.730), ICVF (AUC = 0.815), cellularity (AUC = 0.792) and ADC measurements (AUC = 0.631–0.710) (all *p* < 0.05).

**Conclusion:**

td-dMRI-derived parameters, especially ICVF and cellularity combined with MRI-reported EMVI, show potential as noninvasive biomarkers for LVI prediction in rectal cancer.

**Critical relevance statement:**

This study develops a preoperative time-dependent diffusion MRI-based microstructure parameters model that diagnoses and predicts lymphovascular invasion of rectal cancer, improving diagnostic accuracy and advancing personalized treatment strategies in clinical radiology.

**Key Points:**

- The time-dependent diffusion MRI-derived microstructural parameters model and clinical data for predicting lymphovascular invasion in rectal cancer.
- The combined model outperforms single-modality models with 0.860 AUC and 96.3% specificity.
- The combined model provides a noninvasive, reliable tool for personalized lymphovascular invasion diagnosis and treatment planning.

**Graphical Abstract:**

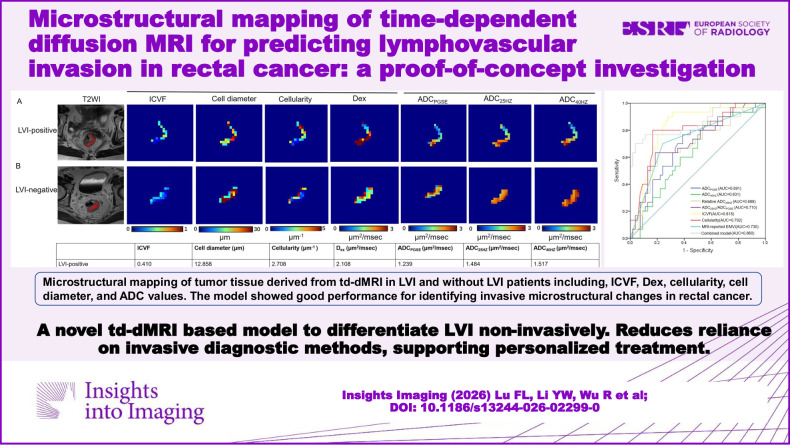

## Introduction

Colorectal cancer is a common malignant tumor, and ranks the second most common cause of cancer-related death [1]. Rectal cancer accounts for one-third of the incidence of colorectal cancer [2]. Lymphovascular invasion (LVI) is characterized by the extension of tumor cells into lymphatic and/or blood [3]. As an important pathological feature of rectal cancer, LVI has been extensively studied and is recognized as an independent prognostic factor for poor outcomes, which is closely associated with a higher risk of death in patients. Previous studies have demonstrated that patients in the LVI-negative group had significantly higher 5-year disease-free survival (71.4% vs 56.2%) and overall survival (86.7% vs 63.4%) rates than those in the LVI-positive group [4, 5]. Therefore, LVI is an important indicator of poor prognosis in rectal cancer, associated with high recurrence and metastatic potential [6, 7]. Accurate preoperative detection of LVI can help clinicians stratify patients by risk and tailor treatments. For example, patients who are LVI-positive require further preoperative neoadjuvant radiochemotherapy to improve prognosis [6]. However, LVI can only be identified by postoperative pathological examination rather than the preoperative imaging method or pathological biopsy.

Several previous studies have developed models comprising radiomics features for noninvasive prediction of LVI in rectal cancer [8–10]. However, radiomics encounter the problem of poor reproducibility of results and biologic interpretation, which would make it hard to translate radiomic models into clinical practice [11, 12]. A previous study reported that conventional apparent diffusivity coefficients (ADC) values provided a noninvasive method for predicting LVI [10]. However, there is an overlap in ADC values between the LVI-positive and LVI-negative groups. A possible explanation could be that ADC is only a simple measure of restricted diffusivity that is affected by several factors, such as inflammation and cell proliferation, and does not offer the ability to identify the microstructural changes that indicate LVI, underscoring the need for advanced imaging methods. Time-dependent diffusion MRI (td-dMRI) enables quantification of microstructural properties such as cell diameter, cellularity, and intracellular volume fraction [13, 14]. td-dMRI has been explored as a promising method in other tumors, such as breast and prostate cancer, but its clinical value in predicting LVI in rectal cancer remains unestablished [15, 16]. Given that LVI is associated with increased tumor invasiveness and cellularity [17, 18], we hypothesized that microstructural parameters derived from td-dMRI could be utilized to predict LVI in rectal cancer. Therefore, the purpose of this study was to evaluate td-dMRI-based microstructural parameters for noninvasive prediction of LVI status in patients with resectable rectal cancer.

## Materials and methods

### Patients

**Fig. 1:**
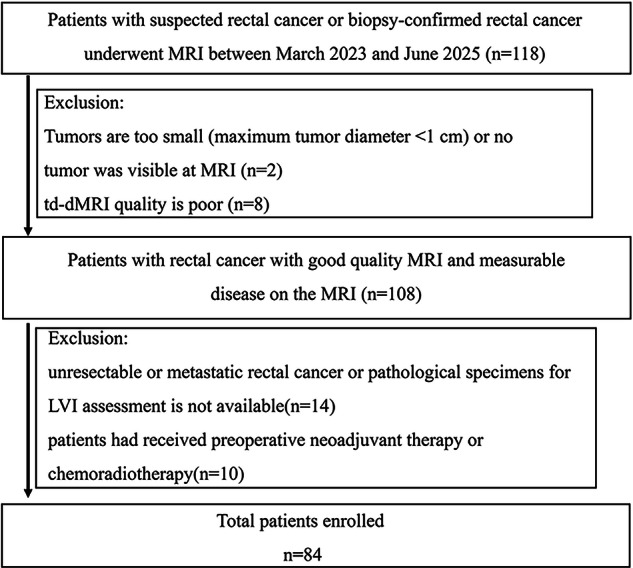
A flowchart of the study participants

This prospective study was approved by the institutional review board, and written informed consent was obtained from all participants. From March 2023 to June 2025, 118 consecutive patients were enrolled based on the following inclusion criteria: (1) clinical suspicious of rectal cancer (a change in bowel habits, bright red blood in stool, abdominal pain) and biopsy-confirmed rectal cancer; (2) tumor located within 15 cm from the anal verge, as determined by endoscopy or imaging; (3) Tumor stages T1, T2, T3a, T3b, and T4a (high-position rectal cancer) [19]; (4) no prior treatment for rectal cancer, including chemotherapy, or radiotherapy; (5) completed td-dMRI examinations before any treatment. The exclusion criteria were as follows: (1) tumors are too small (maximum tumor diameter < 1 cm) or no tumor was visible at MRI (*n* = 2); (2) td-dMRI quality is poor (ie, severe motion artifacts, *n* = 8); (3) unresectable or metastatic rectal cancer or pathological specimens for LVI assessment is not available (*n* = 14); (4) patients had received preoperative neoadjuvant therapy or chemoradiotherapy (*n* = 10). A flowchart of the study participants is described in Fig. 1.

### MR protocol

MRI was performed on a 3.0-T MR scanner (Magneton Vida, Siemens Healthineers) with a 32-channel body coil. The patients were placed in a normative supine position when the respiratory signals were established, and all the patients were given an intramuscular injection of 20 mg of scopolamine butylbromide to reduce bowel motion. Water or air to distend the rectum was not used in this study. Imaging included both oscillating gradient spin-echo (OGSE) and pulsed gradient spin-echo (PGSE) sequences from the Siemens research sequence. OGSE sequences were acquired at oscillating frequencies of 25 Hz (effective diffusion time = 7.3 msec, one cycle, b = 0, 350 and 710 sec/mm^2^) and 40 Hz (effective diffusion time = 4.7 msec, two cycles, b = 0, 150 and 315 sec/mm^2^), and PGSE sequences at diffusion duration and separation of 54.3 and 59.3 msec, respectively (effective diffusion time = 41.1 msec, b = 0, 350, and 710 sec/mm^2^). The following parameters were used for all sequences: three diffusion directions; repetition time (TR) /echo time (TE), 5000/132 msec; field of view, 260 × 265 mm^2^; spacing, 10%; in-plane resolution, 3.2 × 3.2 mm^2^; number of sections, 10; and section thickness, 5 mm. This td-dMRI protocol scanning time was approximately 4.5 min.

Other clinical routine MR sequences including axial-oblique T2-weighted imaging (T2WI) and the scanning orientation of axial-oblique T2WI was perpendicular to the tumor within the patient’s rectal lumen, aiming to clearly visualize the maximum cross-sectional plane of the tumor. The orientations of the OGSE and PGSE sequences were consistent with that of the oblique axial T2WI. Parameters of axial-oblique T2WI: TR/TE = 4600 msec/75 msec, field of view = 220 mm × 220 mm^2^, spacing: 10%; matrix size = 256 × 512, slice thickness = 3 mm, along with sagittal and coronal T2WI. Contrast-enhanced axial T1-weighted imaging (T1WI), 3D fat-saturated gradient-echo (GRE) sequence, TR: 4.6 msec; TE: 2.1 msec; flip angle: 15°; matrix size: 320 × 256; field of view: 280 × 320 mm^2^; spacing: 0%; slices thickness: 1.1 mm. Contrast medium: Gadopentetate Dimeglumine, Bayer Healthcare, 0.1 mmol/kg body weight, 3 mL/s injection rate and 20 mL normal saline (0.9% NaCl) flush. Scanning time: 1 min 48 s.

### Image analysis

Two radiologists (with 5 and 12 years of experience in rectal cancer) who were blinded to the clinical information, working in consensus, reviewed the MR images in 84 patients with rectal cancer. The tumor length and maximal tumor thickness were obtained on the sagittal and oblique T2WI images, respectively. The extramural vascular invasion (EMVI) status of the primary tumor and T stage were also evaluated on (oblique axial T2WI, coronary, and sagittal) T2WI [20]. MRI-reported lymph node status was based on the 2016 European Society of Gastrointestinal and Abdominal Radiology consensus meeting [21].

**Fig. 2:**
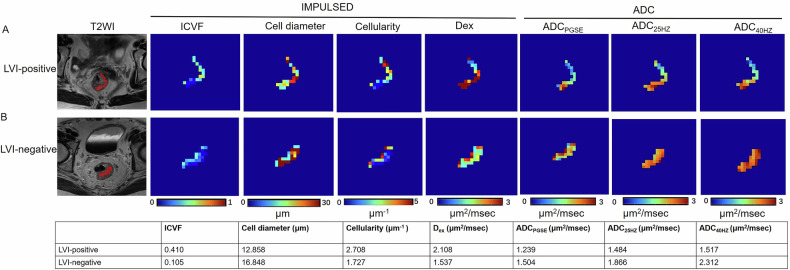
Microstructural mapping of tumor tissue derived from time-dependent diffusion MRI (td-dMRI) in two representative patients: one with lymphovascular invasion (LVI) (**A**) and one without LVI (**B**). Microstructural maps including the intracellular volume fraction (ICVF), cell diameter, cellularity, extracellular diffusivity (Dex), and apparent diffusion coefficient (ADC) at 25 Hz OGSE, 45 Hz OGSE, and PGSE. The patient with LVI shows significantly elevated ICVF and diameter values, with lower ADCs, illustrating the potential of td-dMRI-derived parameters for identifying invasive microstructural changes in tumors

The td-dMRI data were fitted using imaging microstructural parameters with the limited spectrally edited diffusion (IMPULSED) model implemented in MATLAB (MathWorks, Inc.) [22]. Voxel model fitting was performed by a least squares algorithm to generate parametric maps of microstructural features, including intracellular volume fraction (ICVF), cell diameter, extracellular diffusivity (D_ex_) and cellularity. Regions of interest (ROIs) were delineated manually by an experienced radiologist (first author, with 5 years of experience in rectal cancer) on each tumor’s largest cross-sectional area, guided by td-dMRI microstructural maps and ADC images (Fig. 2). ROIs were carefully placed to encompass solid tumor components referring to T2WI and contrast-enhanced T1WI images while excluding surrounding normal tissue, blood vessels, and necrotic areas to minimize confounding effects on parameter estimation. ADC values at different diffusion times were also calculated, including ADC derived from PGSE (ADC_PGSE_), ADC measurement obtained at 25 Hz (ADC_25HZ_), 40 Hz (ADC_40HZ_). Additionally, relative ADC parameters were computed, including relative ADC_25HZ_ ([ADC_25HZ_ − ADC_PGSE_]/ADC_PGSE_) [23], relative ADC_40HZ_ ([ADC_40HZ_ − ADC_PGSE_]/ADC_PGSE_), ADC_25HZ_/ADC_40HZ_ [24], ADC_25HZ_/ADC_PGSE_ [25], and ADC_40HZ_/ADC_PGSE_.

To evaluate the stability and reliability of the microstructural features and ADC values by interobserver intraclass correlation coefficient (ICC) analysis, a radiologist (the second author, with 12 years of experience in rectal cancer) repeated the delineation for the first 30 patients’ images. The mean values of the corresponding measurements of two radiologists were used for further analysis. The ICC and Bland–Altman analysis were calculated to test the inter-rater reliability.

### Histopathologic assessment and correlation with MRI

LVI is defined as the presence of tumor thrombi in the vascular and/or lymphatic wall infiltration or the presence of tumor emboli with an endothelium-lined space. LVI analysis on pathology was performed on all 84 patients in this study. And the diagnosis of LVI was classified according to the 8th American Joint Committee on Cancer Staging (AJCC) system [26].

In 84 patients, twenty patients’ (8 with LVI-positive and 12 with LVI-negative) high-definition (magnification, × 400) hematoxylin and eosin (H&E)-stained sections of rectal cancer tissue were randomly selected to assess the correlation between td-dMRI-based microstructural parameters and pathologic microstructural parameters. All histopathological slides were interpreted by a pathologist (with 15 years of experience in pathology) without knowing the MRI findings. Whole-slide digital tissue sections were processed using Qupath (version 0.6.0) combined with Openslide (version 4.0.0) software, and ROIs were delineated and identified, ensuring at least 20,000 cells were included. Microstructural data were obtained by implementing a cell detection module and applying an automatically quantifying conditional generative adversarial network (CGAN) for nuclear segmentation [27]. This process provided the primary measurements of the whole nuclear area (A_nuclei_) and the total delineated tissue area (A_tissue_) [28]. Subsequently, the following pathologic microstructural parameters, conceptually analogous to those derived from td-dMRI, were calculated: the volume-weighted diameter of the nuclei was calculated as ${{{{\rm{d}}}}}_{{{{\rm{nuclei}}}}}={\sum }_{n}{d}_{n}^{4}/{\sum }_{n}{d}_{n}^{3}$, where *n* represents the cell number, d is diameter, and $\varSigma$ is the sum, and the nucleus diameter (d_nuclei_) was scaled to the cell diameter based on a scaling factor of 1.8 [25]. Pathology-based intracellular fraction was calculated as ${({\sum }_{n}{A}_{{nuclei}}/{A}_{{tissue}})}^{3/2}$, where A_tissue_ is the area of the whole tissue and A_nuclei_ is the area of the whole nuclei. The pathologic cellularity was calculated as follows: (pathologic cellularity = [intracellular fraction pathologic structure/diameter pathologic structure] × 100).

The validation of the pretrained CGAN was performed through a correlation analysis between these quantitatively derived pathologic parameters and the corresponding parameters obtained from td-dMRI. Pearson correlation analysis was used to assess the relationship between td-dMRI-based ICVF and pathologic intracellular fraction, td-dMRI-based cell diameter and pathologic cell diameter, and td-dMRI-based cellularity and pathologic cellularity.

### Statistical analysis

Statistical analyses were performed using SPSS 26.0 (IBM) and MedCalc 15.2. Continuous variables were expressed as mean ± SD and compared by independent *t*-test with normal distribution or Mann–Whitney U test with abnormal distribution. Qualitative variables were compared using the χ^2^ test. Univariable and multivariable logistic regression analyses using backward stepwise selection were performed to identify risk factors for LVI-positive. The area under the receiver operating characteristic curve (AUC) was used to determine diagnostic performance for predicting LVI-positive. The corresponding sensitivity and specificity were also calculated. The cutoff values of the quantitative parameters were determined by maximizing the Youden index. The DeLong test was used to compare the AUCs. The ICC and Bland–Altman analysis were utilized to assess interobserver agreement. ICC < 0.5, 0.5–0.75, 0.75–0.9, > 0.9 indicated poor, moderate, good and excellent reproducibility, respectively. *p* < 0.05 indicates a significant difference.

## Results

### Participant characteristics

A total of 84 participants (median age, 66 years [interquartile range, 59–70 years]) with rectal cancer were enrolled in this study, including 30 participants with LVI-positive status and 54 participants with LVI-negative status. There were no significant differences between the LVI-positive and LVI-negative groups regarding age (*p* = 0.125), gender (*p* = 0.947), tumor location (*p* = 0.182), tumor length (*p* = 0.968), tumor thickness (*p* = 0.085), cT stage (*p* = 0.071), or cN stage (*p* = 0.513) (Table 1). The prevalence of MRI-reported EMVI was significantly higher in the LVI-positive group compared with the LVI-negative group (*p* < 0.001). No significant differences were observed in pre-surgery CEA levels (*p* = 0.973) or CA199 levels (*p* = 0.680) between the two groups.

**Table 1:** Baseline characteristics stratified by LVI status

Characteristic	LVI-positive (*n* = 30)	LVI-negative (*n* = 54)	*p*-value
Age (years)	67 (60–70)	62 (57–70)	0.125
Gender			0.947
Male	18 (60%)	32 (55.1%)	
Female	12 (40%)	22 (44.9%)	
Location			0.182
10–15 cm	0 (0)	5 (9.3%)	
5–10 cm	13 (43.3%)	25 (46.3%)	
< 5 cm	17 (56.7%)	24 (44.4%)	
Tumor length (cm)	5.34 ± 1.99	5.26 ± 2.13	0.968
Tumor thickness	2.02 ± 0.92	1.71 ± 0.57	0.085
pT stage			0.071
T1, T2	1 (3.3%)	9 (16.7%)	
T3a, T3b, and T4a	29 (96.7%)	45 (83.3%)	
pN stage			0.513
Negative	15 (50%)	23 (43.6%)	
Positive	15 (50%)	31 (56.4%)	
EMVI			< 0.001
Negative	9 (30%)	41 (75.9%)	
Positive	21 (70%)	13 (24.1%)	
Pre-surgery CEA			0.973
< 5 ng/mL	19 (63.3%)	34 (63%)	
> 5 ng/mL	11 (36.7%)	20 (37%)	
Pre-surgery CA199			0.680
< 35 ng/mL	28 (93%)	49 (90.7%)	
> 35 ng/mL	2 (7%)	5 (9.3%)	

*CEA* carcinoembryonic antigen, *CA199* carbohydrate antigen-199, *pT stage* pathological tumor stage, *pN stage* pathological nodal stage, *EMVI* extramural venous invasion, *LVI* lymphovascular invasion

### The correlation between td-dMRI microstructural parameters and LVI

**Fig. 3:**
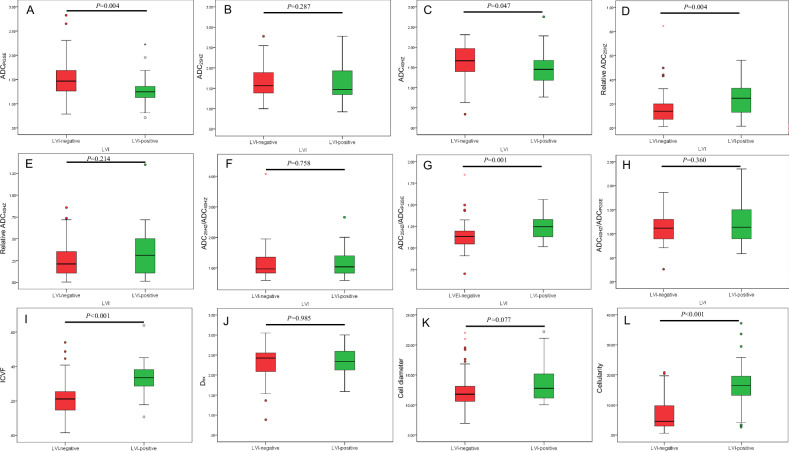
Box plots show comparisons of ADC_PGSE_ (**A**), ADC_25HZ_ (**B**), ADC_40HZ_ (**C**), relative ADC_25HZ_ (**D**), relative ADC_40HZ_ (**E**), ADC_25HZ_/ADC_40HZ_ (**F**), ADC_25HZ_/ADC_PGSE_ (**G**), ADC_40HZ_/ADC_PGSE_ (**H**), intracellular volume fraction (ICVF) (**I**), Dex (**J**), cell diameter (**K**), and cellularity (**L**) between LVI-positive and LVI-negative. Significant differences are observed in ICVF, cell diameter, and several ADC-related parameters, with higher ICVF, cell diameter and cellularity in LVI-positive

There were no significant differences for ADC_25HZ_, Relative ADC_40HZ_, ADC_25HZ_/ADC_40HZ_, ADC_40HZ_/ADC_PGSE_, D_ex_, and cell diameter between patients with LVI-positive and LVI-negative (all *p* > 0.05) (Table 2). The proportion of EMVI-positive in the LVI-positive group was higher than that in the LVI-negative group (*p* < 0.001). The ADC_PGSE_ (*p* = 0.004) and ADC_40HZ_ (*p* = 0.047) were lower in the LVI-positive group than in the LVI-negative group. Relative ADC_25HZ_ (*p* = 0.004), ADC_25HZ_/ADC_PGSE_ (*p* = 0.001), ICVF (*p* < 0.001), and cellularity (*p* < 0.001) were higher in the LVI-positive group than in the LVI-negative group (Fig. 3).

**Table 2:** The correlation between time-dependent diffusion MRI-derived microstructural parameters and LVI

Td-dMRI microstructural parameters	LVI-positive (*n* = 30)	LVI-negative (*n* = 54)	*p*-value
ADC_PGSE_	1.273 ± 0.317	1.497 ± 0.401	0.004
ADC_25HZ_	1.583 ± 0.420	1.688 ± 0.455	0.287
ADC_40HZ_	1.450 (1.167-1.681)	1.664 (1.390–1.977)	0.047
Relative ADC_25HZ_	0.247 (0.129–0.333)	0.140 (0.067–0.202)	0.004
Relative ADC_40HZ_	0.309 (0.107–0.514)	0.212 (0.105–0.352)	0.214
ADC_25HZ_/ADC_40HZ_	1.037 (0.830–1.404)	0.967 (0.828–1.353)	0.758
ADC_25HZ_/ADC_PGSE_	1.247 (1.129–1.333)	1.134 (1.045–1.198)	0.001
ADC_40HZ_/ADC_PGSE_	1.138 (0.887–1.514)	1.116 (0.891–1.307)	0.360
ICVF	0.334 (0.282–0.382)	0.212 (0.145–0.261)	< 0.001
D_ex_	2.341 (2.120–2.611)	2.425 (2.079–2.561)	0.985
Cell diameter	12.808 (11.133–15.195)	11.812 (10.600–13.293)	0.077
Cellularity	2.861 (2.443–3.313)	1.964 (1.375–2.579)	< 0.001

*Td-dMRI* time-dependent diffusion MRI, *LVI* lymphovascular invasion, *ICVF* intracellular volume fraction, *D*_*ex*_ extracellular diffusivity, *ADC*_*PGSE*_, PGSE-based ADC, *ADC*_*25HZ*_ ADC measurement at 25 Hz, *ADC*_*40HZ*_ ADC measurement at 40 Hz

### Risk factors for the prediction of LVI positivity

Univariable analysis showed that EMVI (odds ratio (OR), 7.359 [95% CI: 2.709, 19.993], *p* < 0.001), ADC_PGSE_ (OR, 0.344 [95% CI: 0.135, 0.874], *p* = 0.025), relative ADC_25HZ_ (OR, 2.917 [95% CI: 1.131, 7.523], *p* = 0.027), ADC_25HZ_/ADC_PGSE_ (OR, 2.916 [95% CI: 1.128, 7.526], *p* = 0.028), ICVF (OR, 13.000 [95% CI: 4.3, 40.234], *p* < 0.001), and cellularity (OR, 1.190 [95% CI: 1.099, 1.289], *p* < 0.001) were associated with LVI-positive. Multivariable logistic regression analysis identified EMVI (OR, 3.251 [95% CI: 0.991–10.699], *p* = 0.032), ICVF (OR, 8.137 [95% CI: 2.836, 27.750], *p* = 0.001), and cellularity (OR, 1.159 [95% CI: 1.062, 1.264], *p* = 0.001) as independent risk factors for predicting LVI-positive (Table 3).

**Table 3:** Univariate and multivariate logistic regression analysis for clinical characteristics and microstructural parameters for predicting lymphovascular invasion in rectal cancer

Parameters	Univariate analysis		*p*-value	Multivariate analysis		*p*-value
	OR	95% CI		OR	95% CI	
Gender	0.939	(0.824, 1.070)	0.125			
Age	1.031	(0.415, 2.562)	0.947			
Location	0.753	(0.476, 1.193)	0.354			
cT stage	1.253	(0.204, 7.714)	0.190			
Pathological LN	0.366	(0.112–1.193)	0.623			
MRI-reported EMVI	7.359	(2.709, 19.993)	< 0.001	3.251	(0.991, 10.699)	0.032
CA199	1.027	(0.899, 1.172)	0.272			
CEA	0.951	(0.871, 1.040)	0.878			
CA242	0.994	(0.927–1.066)	0.237			
Tumor length	1.108	(0.820, 1.263)	0.870			
Wall thickness	1.214	(0.181, 8.123)	0.776			
ADC_PGSE_	0.344	(0.135, 0.874)	0.025	0.144	(0.006, 3.587)	0.238
ADC_25HZ_	2.168	(0.102, 45.847)	0.287			
ADC_40HZ_	0.612	(0.249, 1.504)	0.284			
Relative ADC_25HZ_	2.917	(1.131, 7.523)	0.027	7.536	(0.257, 220.842)	0.241
Relative ADC_40HZ_	0.465	(0.053, 4.063)	0.214			
ADC_25HZ_/ADC_40HZ_	1.145	(0.030, 44.457)	0.758			
ADC_25HZ_/ADC_PGSE_	2.916	(1.128, 7.526)	0.028	0.329	(0.010, 10.715)	0.531
ADC_40HZ_/ADC_PGSE_	1.683	(0.024, 11.658)	0.604			
ICVF	13.000	(4.3, 40.234)	< 0.001	8.137	(2.836, 27.750)	0.001
D_ex_	1.905	(0.115, 31.566)	0.727			
Cell diameter	1.287	(0.945, 1.753)	0.167			
Cellularity	1.190	(1.099, 1.289)	< 0.001	1.159	(1.062, 1.264)	0.001

*ICVF* intracellular volume fraction, *D*_*ex*_ extracellular diffusivity, *LN* lymph node, *EMVI* extramural vascular invasion, *CEA* carcinoembryonic antigen, *CA199* carbohydrate antigen-199, *CA242* carbohydrate antigen-242, *ADC*_*PGSE*_ PGSE-based ADC, *ADC*_*25HZ*_ ADC measurement at 25 Hz, *ADC*_*40HZ*_ ADC measurement at 40 Hz

### Diagnostic performance of risk models

**Fig. 4:**
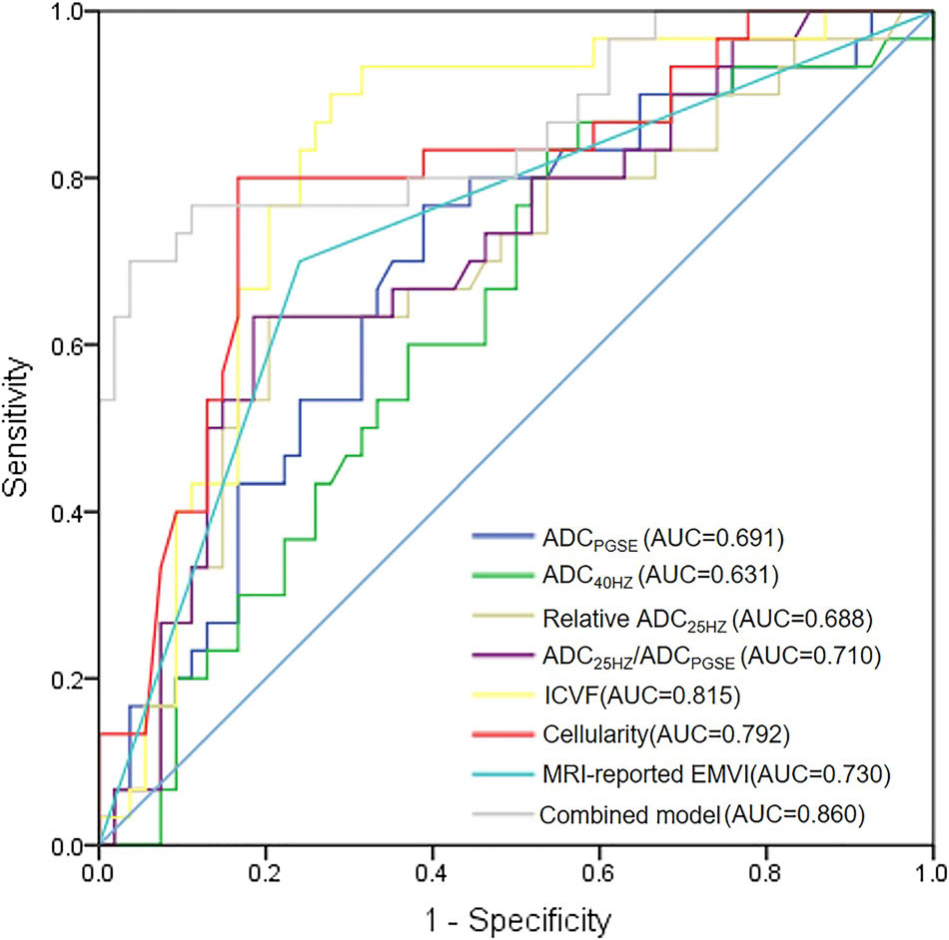
Receiver-operating characteristic curves illustrating the diagnostic performance of various time-dependent diffusion MRI (td-dMRI) parameters, including ADC_PGSE_, ADC_40HZ_, relative ADC_25HZ_, ADC_25HZ_/ADC_PGSE_, intracellular volume fraction (ICVF), cellularity, and MRI-reported extramural vascular invasion (EMVI), along with a combined model (ICVF, cellularity, and EMVI), for predicting lymphovascular invasion. The combined model achieved the highest AUC (0.860), highlighting their potential as a robust predictor of invasive tumor characteristics

The diagnostic performance of individual risk factors and the combined model is summarized in Table 4. Radiological feature constructed by MRI-reported EMVI achieved an AUC of 0.730 (95% CI: 0.613, 0.846) for predicting LVI-positive. ICVF demonstrated superior predictive performance (AUC, 0.815 [95% CI: 0.710–0.888]) compared with ADC_PGSE_ (AUC, 0.691 [95% CI: 0.580–0.787], *p* < 0.010), ADC_40HZ_ (AUC, 0.631 [95% CI: 0.510–0.753], *p* = 0.003), relative ADC_25HZ_ (AUC, 0.688 [95% CI: 0.581–0.780], *p* = 0.101), ADC_25HZ_/ADC_PGSE_ (AUC, 0.710 [95% CI: 0.605–0.808], *p* = 0.001), cellularity (AUC, 0.792 [95% CI: 0.690–0.873], *p* = 0.798). The combined model integrating EMVI, ICVF, and cellularity further improved diagnostic performance, achieving an AUC of 0.860 (95% CI: 0.767–0.926), which was significantly higher than that of MRI-reported EMVI alone (*p* = 0.001) and cellularity alone (*p* = 0.021) (Fig. 4). In pT3a and pT3b rectal cancer subgroups, there were 36 patients with LVI-negative and 23 patients with LVI-positive. For differentiating LVI-positive from LVI-negative in the pT3a and pT3b subgroups, the combined model achieved a good performance with an AUC of 0.834, sensitivity of 69.5%, and specificity of 97.2%.

**Table 4:** Diagnostic performance of time-dependent diffusion MRI-derived microstructural parameters and ADC for predicting lymphovascular invasion in rectal cancer

Parameters	Cutoff	AUC (95% CI)	Sensitivity	Specificity
Radiological feature*	…	0.730 (0.613–0.846)	70.0%	75.9%
ADC_PGSE_ (μm^2^/msec)	1.360	0.691 (0.580–0.787)	76.8%	61.1%
ADC_40HZ_ (μm^2^/msec)	1.559	0.631 (0.510–0.753)	60.0%	61.1%
Relative ADC_25HZ_	0.213	0.688 (0.581–0.788)	63.3%	79.6%
ADC_25HZ_/ADC_PGSE_	1.211	0.710 (0.605–0.808)	63.3%	81.4%
ICVF	0.232	0.815 (0.710–0.888)	90%	70.1%
Cellularity (μm^−1^)	2.329	0.792 (0.690–0.873)	80%	83.3%
Combined model**	0.495	0.860 (0.767–0.926)	70.0%	96.3%

*ICVF* intracellular volume fraction, *Dex* extracellular diffusivity, *ADC*_*25HZ*_ ADC measurement at 25 Hz, *ADC*_*40HZ*_ ADC measurement at 40 Hz

* Radiological feature was constructed by MRI-reported extramural vascular invasion

** Combined model was based on MRI-reported extramural vascular invasion, ICVF, and cellularity

### Validation with histopathologic findings

**Fig. 5:**
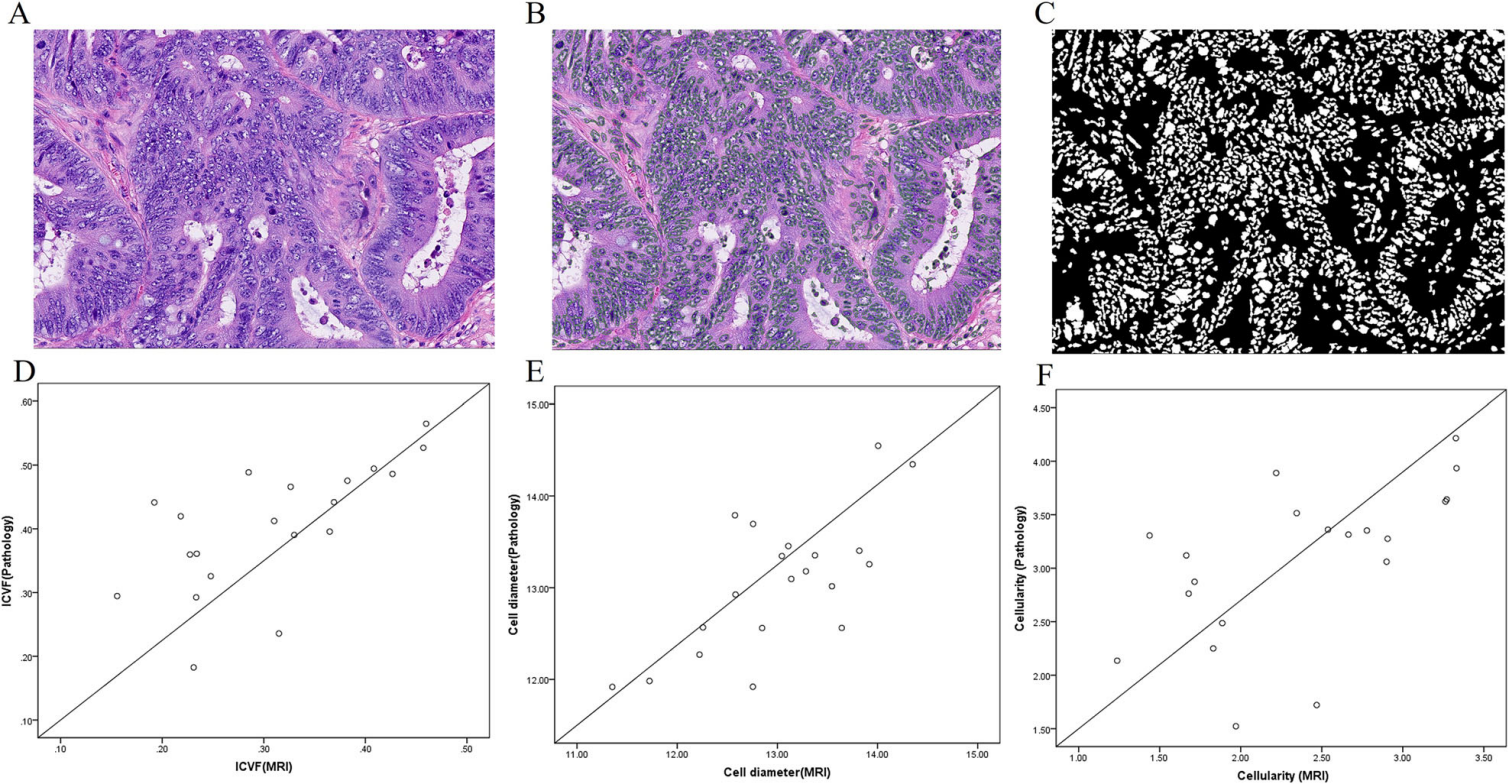
The correlations between time-dependent diffusion MRI (td-dMRI)-based microstructural parameters and results of pathologic examination-based microstructural properties. **A** Hematoxylin-eosin-stained image (magnification, × 400) shows pathologic specimens from one patient. **B** The nuclei in each whole-slide image were segmented by using a pretrained conditional generative adversarial network. **C** The pathologic microstructural properties were automatically quantified. The correlation between td-dMRI-based intracellular volume fraction (ICVF) (**D**), cell diameter (**E**), and cellularity (**F**) and pathologic examination-based microstructural properties

The nuclei in each whole-slide image were segmented by using a pretrained CGAN (Fig. 5). The results indicated that there was a high correlation between td-dMRI-based ICVF and pathologic ICVF (*r* = 0.671, *p* = 0.001), and td-dMRI-based cell diameter (*r* = 0.736, *p* < 0.001) and pathologic cell diameter. There was a moderate correlation between td-dMRI-based cellularity and pathologic cellularity (*r* = 0.590, *p* = 0.006).

### Interobserver agreement

**Fig. 6:**
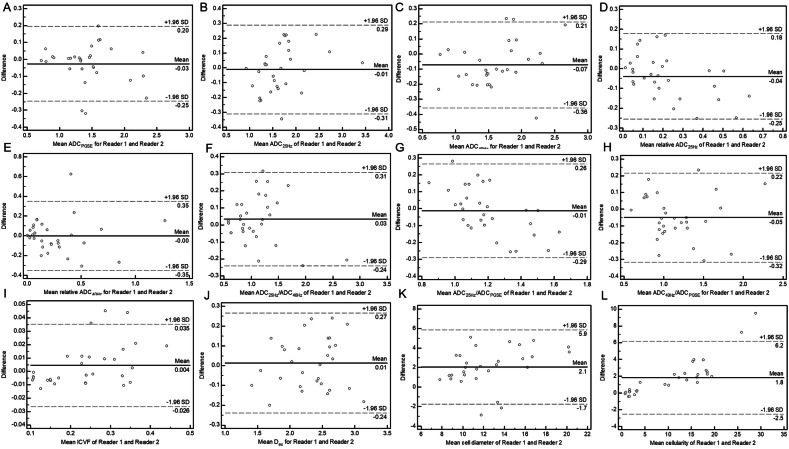
Bland–Altman plots compare the reproducibility of MRI microstructural parameter measurements and apparent diffusion coefficient (ADC) value measurements at different diffusion times from two independent readers. **A** There was excellent agreement for mean ADC_PGSE_ (ICC=0.965. 95% CI: 0.928–0.983); **B** there was excellent agreement for mean ADC_25HZ_(ICC=0.957, 95% CI: 0.911–0.980); **C** there was excellent agreement for mean ADC_40HZ_ (ICC=0.951, 95% CI: 0.900–0.976); **D** there was good agreement for mean relative ADC_25HZ_ (ICC=0.813, 95% CI: 0.645–0.907); **E** there was good agreement for mean relative ADC_40HZ_ (ICC=0.817, 95% CI: 0.650–0.908); **F** there was excellent agreement for mean ADC_25HZ/40HZ_(ICC=0.951, 95% CI: 0.900–0.977); **G** there was good agreement for mean ADC_25HZ_/ADC_PGSE_(ICC=0.764, 95% CI: 0.561–0.880); **H** there was excellent agreement for mean ADC_40HZ_/ADC_PGSE_ (ICC=0.936, 95% CI: 0.869–0.969); **I** there was excellent agreement for mean intracellular volume fraction (ICVF) (ICC=0.986, 95% CI: 0.970–0.993); **J** there was excellent agreement for mean *D*_ex_ (ICC=0.955, 95% CI: 0.908-0.978); **K**. there was good agreement for mean cell diameter (ICC=0.839, 95% CI: 0.689–0.920); **L** there was excellent agreement for mean cellularity (ICC=0.963, 95% CI: 0.925–0.982)

Interobserver agreement for the measurements of microstructural features and ADC values is shown in Table 5. The ICC values for relative ADC_25HZ_, relative ADC_40HZ_, ADC_25HZ_/ADC_PGSE_, and cell diameter were 0.764–0.839 (95% CI: 0.561–0.920), indicating good agreement, and for other parameters were 0.936–0.986 (95% CI: 0.869–0.993), indicating excellent agreement. Bland–Altman analysis is shown in Fig. 6.

**Table 5:** Interobserver agreement for various parameters

Parameters	Intraclass coefficient correlation (95% CI)
ADC_PGSE_	0.965 (0.928–0.983)
ADC_25HZ_	0.957 (0.911–0.979)
ADC_40HZ_	0.951 (0.900–0.976)
Relative ADC_25HZ_	0.813 (0.645–0.907)
Relative ADC_40HZ_	0.817 (0.650–0.908)
ADC_25HZ_/ADC_40HZ_	0.951 (0.900–0.977)
ADC_25HZ_/ADC_PGSE_	0.764 (0.561–0.880)
ADC_40HZ_/ADC_PGSE_	0.936 (0.869–0.969)
ICVF	0.986 (0.970–0.993)
D_ex_	0.955 (0.908–0.978)
Cell diameter	0.839 (0.689–0.920)
Cellularity	0.963 (0.925–0.982)

*ICVF* intracellular volume fraction, *D*_*ex*_ extracellular diffusivity, *ADC*_*PGSE*_ PGSE-based ADC, *ADC*_*25HZ*_, ADC measurement at 25 Hz, *ADC*_*40HZ*_ ADC measurement at 40 Hz

## Discussion

In this study, we demonstrated that microstructural parameters derived from td-dMRI, particularly ICVF and cellularity, are valuable noninvasive biomarkers for predicting LVI in rectal cancer. Multivariable analysis indicated that EMVI (OR, 3.251), ICVF (OR, 8.137), and cellularity (OR, 1.159) were independent risk factors for predicting LVI-positive. The combined model integrating these features achieved the highest diagnostic performance, with an area under the receiver operating characteristic curve (AUC) of 0.860. In pT3a and pT3b rectal cancer subgroups, the combined model achieved a good performance with an AUC of 0.834.

Conventional DWI, which allows quantification of diffusion-driven displacement of water molecules, has been utilized to predict LVI in rectal cancer. Chen et al found that DWI-based gross tumor volume showed the most potential for predicting LVI with an AUC of 0.899 [29]. Nonetheless, tumor volume measurements on MRI can be time-consuming, which makes it difficult to translate to clinical practice. Yuan et al indicated the ratio of peritumoral ADC to intratumoral ADC showed better predictive performance for assessing LVI with an AUC of 0.778 [30]. Fan et al used pretreatment ADC mapping to predict LVI and achieved an AUC ranging from 0.71 to 0.80 [31]. In this study, we found ADC_PGSE_ could help predict LVI with an AUC of 0.691. However, Curvo-Semedo et al reported that ADC could not help to assess the LVI in patients with rectal cancer [32]. These studies indicated the value of ADC for predicting LVI remains controversial. Furthermore, we found OGSE yields higher ADC values (ADC_25HZ_ and ADC_40HZ_) than those obtained from PGSE (ADC_PGSE_). The explanation could be that ADC value may decrease with a longer diffusion time, as more water collides with cell membranes [33]. Therefore, we assumed the relative changes in ADC values with different diffusion times may offer new information for describing tumor microstructure. We found that LVI-positive patients had higher relative ADC_25HZ_ than LVI-negative patients. This finding may indicate that patients with LVI-positive have more microstructures with hindered diffusion than those of LVI-negative. We also found a significant correlation between ADC_25HZ_/ADC_PGSE_ and LVI-positive. Compared with ADC_OGSE_ or ADC_PGSE_, ADC_25HZ_/ADC_PGSE_ tended to have a slightly higher AUC for predicting LVI-positive. This finding may indicate that ADC_25HZ_/ADC_PGSE_ better reflects tumor microstructures than ADC_PGSE_, which was similar to the Ejima et al observation in endometrial cancer [24]. Despite the potential ability of ADC measurements for predicting LVI-positive, the diagnostic performance (AUC, 0.631–0.710) was still unsatisfactory. Artificial intelligence analysis methods showed excellent predictive performance for predicting LVI (AUC, 0.876–0.913) [34, 35]. However, the AUCs of radiomics models are questionable due to flexible algorithms, raising concerns about overfitting [12]. Additionally, lacking biophysical support and interpretability make it difficult to translate to clinical practice.

The IMPULSED model, based on a two-compartment model, simplifies mathematical calculations by considering cancer cell spheres. The clinical feasibility of the IMPULSED method has been explored in breast cancer, and cellularity achieved a high AUC of 0.84 for predicting pathologic complete response [16]. Cao et al utilized the IMPULSED method and found ICVF achieved a high AUC of 0.936 for differentiating high-grade serous ovarian cancer from serous borderline ovarian tumor [36]. In this study, we demonstrated that ICVF and cellularity are two crucial parameters among the td-dMRI microstructural parameters for predicting LVI-positive, which was superior to that of ADC measurement and radiological feature. The explanation could be that there is an association between LVI-positive and poor differentiation, and poorly differentiated tumors contain a large nuclear volume fraction, increased cell density, and micronecrosis [37]. Therefore, microstructures in poorly differentiated tumors have higher complexity than those in well-differentiated tumors. We may presume that higher cellularity and ICVF in tumors are associated with increased proliferation and invasiveness, which result in higher LVI-positive. Compared with a radiological feature and cellularity, a combined model integrating MRI-reported EMVI, ICVF, and cellularity achieved a better predictive performance (AUC, 0.730 vs 0.792 vs 0.860, all *p* < 0.05) and higher specificity (75.9% vs 83.3% vs 96.3%). However, a combined model achieved similar diagnostic performance with ICVF (AUC, 0.860 vs 0.815, *p* = 0.446) but with higher specificity (96.3% vs 83.3%). This finding may indicate that adding MRI microstructural features to MRI-reported EMVI has high potential for wide application for predicting LVI in clinical practice.

There were some limitations in this study. First, the sample size was relatively small in this study. The results were preliminary and need to be verified by additional studies performed in more institutions. Second, MRI microstructural parameters were measured on each tumor’s largest cross-sectional area. Further study with the whole tumor analysis should be performed. Third, although we attempted to correlate td-dMRI parameters with pathologic microstructural features, the pathologic assessment was limited to a selected region from a single slide, which may not precisely correspond to the larger ROI analyzed on MRI due to inherent tumor heterogeneity. This spatial mismatch could introduce bias in the correlation analysis. Future studies incorporating artificial intelligence for whole-tumor volumetric analysis and advanced co-registration techniques between MRI and histopathological whole-slide images are warranted to achieve more precise and comprehensive validation. Moreover, the correlation between td-dMRI parameters and histopathologic microstructure was performed in a relatively small subset of patients (*n* = 20). While the results provide initial proof-of-concept biological validity, future studies with larger sample sizes are warranted for definitive validation. Finally, patients with T3a/b-T4a rectal cancer did not receive preoperative chemoradiotherapy. However, previous studies reported that preoperative chemoradiotherapy has not been shown to significantly increase the 3-year recurrence-free survival rate compared with surgery alone [38, 39].

In conclusion, this study employed td-dMRI-based microstructural mapping to noninvasively predict LVI in patients with rectal cancer and demonstrated that ICVF and cellularity achieved good performance. The combination of ICVF, cellularity, and EMVI further improved the prediction of LVI. Our study should be interpreted as a proof-of-concept demonstrating the feasibility of td-dMRI microstructural mapping for a noninvasive prediction of LVI. In the future, we plan to conduct a multicenter follow-up study to validate our findings.

## Data Availability

The data supporting the results reported in this article can be obtained from the corresponding author upon reasonable request.

## Abbreviations

ADC: Apparent diffusivity coefficients
ADC25HZ: ADC measurement at 25 Hz
ADC40HZ: ADC measurement at 40 Hz
ADCPGSE: PGSE-based ADC
AUC: Area under the ROC curve
Dex: Extracellular diffusivity
EMVI: Extramural vascular invasion
ICC: Intraclass correlation coefficient
ICVF: Intracellular volume fraction
IMPULSED: Imaging microstructural parameters with the limited spectrally edited diffusion
LVI: Lymphovascular invasion
OGSE: Oscillating gradient spin-echo
PGSE: Pulsed gradient spin-echo
ROC: Receiver operating characteristic curve
ROIs: Regions of interests
T1WI: T1-weighted imaging
T2WI: T2-weighted imaging
Td-dMRI: Time-dependent diffusion MRI
